# Respiratory function during enzyme replacement therapy in late-onset Pompe disease: longitudinal course, prognostic factors, and the impact of time from diagnosis to treatment start

**DOI:** 10.1007/s00415-020-09936-8

**Published:** 2020-06-10

**Authors:** David W. Stockton, Priya Kishnani, Ans van der Ploeg, Juan Llerena, Matthias Boentert, Mark Roberts, Barry J. Byrne, Roberto Araujo, Sonia S. Maruti, Nathan Thibault, Karien Verhulst, Kenneth I. Berger

**Affiliations:** 1 Division of Genetic, Genomic and Metabolic Disorders, Departments of Pediatrics and Internal Medicine, Wayne State University and Children’s Hospital of Michigan, Detroit, MI USA; 2 Division of Medical Genetics, Department of Pediatrics, Duke University Medical Center, Durham, NC USA; 3 Center for Lysosomal and Metabolic Diseases, Erasmus MC, University Medical Center, Rotterdam, The Netherlands; 4 Departamento de Genética Médica, Instituto Fernandes Figueira (FIOCRUZ), Rio de Janeiro RJ, Brazil; 5 Department of Neurology, University Hospital of Münster, Münster, Germany; 6 Salford Royal NHS Foundation Trust, Salford, UK; 7 Department of Pediatrics, University of Florida, Gainesville, FL USA; 8 Sanofi Genzyme, Cambridge, MA USA; 9 Sanofi Genzyme, Amsterdam, The Netherlands; 10 Division of Pulmonary, Critical Care and Sleep Medicine, New York University School of Medicine, and the André Cournand Pulmonary Physiology Laboratory, Bellevue Hospital, New York, NY USA

**Keywords:** Pompe disease, Late-onset Pompe disease, Respiratory function, Enzyme replacement therapy, Alglucosidase alfa, Registry

## Abstract

**Objective:**

To examine respiratory muscle function among late-onset Pompe disease (LOPD) patients in the Pompe Registry (NCT00231400/Sanofi Genzyme) during enzyme replacement therapy (ERT) with alglucosidase alfa by assessing the longitudinal course of forced vital capacity (FVC), prognostic factors for FVC, and impact of time from diagnosis to ERT initiation.

**Methods:**

Longitudinal FVC data from LOPD (symptom onset > 12 months or ≤ 12 months without cardiomyopathy) patients were analyzed. Patients had to have baseline FVC (percent predicted upright) assessments at ERT start and ≥ 2 valid post-baseline assessments. Longitudinal analyses used linear mixed-regression models.

**Results:**

Among 396 eligible patients, median baseline FVC was 66.9% (range 9.3–126.0). FVC remained stable during the 5-year follow-up (slope = − 0.17%, *p* = 0.21). Baseline FVC was lower among various subgroups, including patients who were male; older at ERT initiation; had a longer duration from symptom onset to ERT initiation; and had more advanced disease at baseline (based on respiratory support use, inability to ambulate, ambulation device use). Age at symptom onset was not associated with baseline degree of respiratory dysfunction. Differences between subgroups observed at baseline remained during follow-up. Shorter time from diagnosis to ERT initiation was associated with higher FVC after 5 years in all patients and the above subgroups using a cut-off of 1.7 years.

**Conclusion:**

FVC stability over 5 years suggests that respiratory function is preserved during long-term ERT in real-world settings. Early initiation of alglucosidase alfa was associated with preservation of FVC in LOPD patients with better respiratory function at the time of treatment initiation.

## Introduction

Pompe disease is a rare, autosomal recessive, progressive neuromuscular disorder caused by deficient lysosomal acid α-glucosidase (GAA), an enzyme that breaks down glycogen. Resulting abnormal accumulation of lysosomal glycogen leads to cellular dysfunction; progressive respiratory, cardiac, skeletal, and smooth muscle damage; and functional disabilities. While the majority of patients exhibit progressive muscle weakness, Pompe disease presents as a broad clinical spectrum with considerable variation in age at symptom onset, presenting signs and symptoms, severity, organ involvement, and rate of progression. In late-onset Pompe disease (LOPD), symptoms can present at any age, but typically without cardiomyopathy during early childhood. Although Pompe disease affects multiple systems, respiratory muscle dysfunction and failure are sources of significant morbidity and mortality in LOPD [1–4].

Enzyme replacement therapy (ERT) with alglucosidase alfa has been approved for the treatment of Pompe disease since 2006 [5, 6]. Relatively few studies have examined the association between the time of initiation of therapy and long-term ERT with alglucosidase alfa with outcomes in LOPD patients. Alglucosidase alfa has been shown to improve and stabilize skeletal and respiratory muscle function within the first 2 years of treatment [1, 7, 8]. A more recent study reported that after 5 years of alglucosidase alfa treatment, muscle strength, respiratory muscle function, walking distance, and daily life activities showed improvement and often stability compared to baseline values and the expected natural course of the disease [9].

Among LOPD patients treated with alglucosidase alfa, the timing of treatment initiation in relation to onset of symptoms of Pompe disease and diagnosis is of particular interest. With increased awareness among the healthcare community, recent initiatives such as newborn screening, and advances in diagnostic technologies, the time between symptom onset and diagnosis of Pompe disease may be shortened.

The present study reports on data collected in the Pompe Registry to assess the course of respiratory function in a large multinational cohort of LOPD patients receiving ERT with alglucosidase alfa. Based on previously published results of pulmonary function in LOPD patients during treatment with alglucosidase alfa [7–9], there is a need for further understanding in a large LOPD population. Thus, the objectives of this study were to assess the (1) course of FVC (as percent predicted of normal) during ERT with alglucosidase alfa among LOPD patients in the Pompe Registry, (2) patient characteristics associated with FVC (described henceforth as prognostic factors), and (3) impact of time from diagnosis of Pompe disease to initiation of treatment on the course of FVC.

## Methods

### The Pompe Registry

The Pompe Registry is an ongoing, long-term, multinational, observational program (NCT00231400) designed to improve understanding of the natural history and outcomes of patients with Pompe disease. Started in 2004, the Registry is sponsored and administered by Sanofi Genzyme (Cambridge, MA). Patients with a confirmed diagnosis of Pompe disease can be enrolled by physician investigators worldwide regardless of patient age, clinical manifestations, treatment status, or prior participation in a clinical trial.

Clinical and demographic data for enrolled patients are entered into the Registry by participating healthcare teams. While Registry sites are encouraged to enter all available longitudinal data, the nature and timing of assessments vary as they are influenced by the patient needs, regional practices, resources, and capabilities.

Each independent site is responsible for obtaining patients’ informed written consent to submit their health information to the Pompe Registry, and to use and disclose this information in aggregate analyses. The Registry protocol, informed consent form, and any locally required authorization documents to send patient information to the Registry are reviewed and approved by the local fully constituted Institutional Review Board (IRB) or Independent Ethics Committee (IEC) unless the site provides the Registry with documentation that approval is not required or has been waived by a particular IRB/IEC.

Longitudinal data for upright FVC (expressed as percent of predicted) as of December 2018 from the Pompe Registry were analyzed. The following selection criteria were applied to obtain the final analysis population: enrollment in the Registry; a reported confirmed diagnosis (that is, documented GAA deficiency from any tissue and/or documentation of two pathogenic *GAA* variants) of LOPD, defined as symptom onset at ≤ 12 months of age without cardiomyopathy or symptom onset at > 12 months of age (patients with infantile Pompe disease with cardiomyopathy were excluded); valid FVC administered for patients ≥ 5 years of age and a baseline FVC value < 150%, and reported FVC measurement at baseline with ≥ 2 reported follow-up FVC assessments over a minimal duration of 6 months, as defined below. Exclusion criteria included use of invasive respiratory support at baseline and implausibly low or high longitudinal change in FVC per year, defined as a slope ≤ − 10% per year or ≥  + 10% per year as determined from mixed linear models. The incidence of respiratory support also was assessed.

The baseline period for FVC and clinical measurements was defined as 180 days prior to ERT initiation or up to 28 days after start of treatment. If a patient had multiple values in the baseline period, the record closest to ERT initiation was chosen. Because many patients are enrolled in the Registry after initiation of alglucosidase alfa, some baseline data for respiratory support and ambulation device use were not entered within the baseline window. If data were not entered in the defined baseline period for respiratory support or ambulation device use, the following algorithm was applied: if all records before and after the baseline window were reported as "No", then baseline was also considered "No"; once a patient was reported to have wheelchair use, this was considered to be permanent at all following timepoints. The Pompe Registry Case Report Form (CRF) allows for multiple types of respiratory support to be selected. If multiple types were selected, then the most severe value was used for this analysis (invasive, then non-invasive, then supplemental oxygen, then unknown). The CRF also allows for multiple ambulation devices to be selected. If multiple types were selected, then the most severe value was used (wheelchair, then walker, then crutches, then cane, then other, then unknown).

Follow-up time was defined from the start of ERT to treatment interruption (> 1 month in duration but not permanent) or permanent discontinuation. The maximum duration of follow-up was 5 years after treatment initiation (see “Study design considerations”). FVC was analyzed over time for the total included population and for patients grouped by different baseline FVC classifications as follows: ≤ 55%predicted; > 55 to < 80%predicted; or ≥ 80%predicted. These classifications were based on mean baseline FVC values of treated patients reported in previous Registry publications and published guidelines for spirometry testing [7, 10, 11]. We also compared the impact of a shorter versus longer time from diagnosis to start of alglucosidase alfa. Groups were defined by the median time from diagnosis to treatment initiation (1.7 years): Shorter-Time (0–1.7 years, *n* = 198) versus Longer-Time (> 1.7 years, *n* = 198) for hypothesis testing, with sensitivity analysis in which time from diagnosis to treatment initiation was examined in tertiles and as a continuous measure. These groups were defined based on the time from diagnosis to time of ERT initiation and not patient age.

### Study design considerations

Upright FVC % predicted during alglucosidase alfa treatment was the outcome selected as it is the respiratory measurement most widely used in clinical practice. In addition, it has been used as a co-primary endpoint in other LOPD studies [7, 8]. The chosen maximum duration of follow-up was 5 years after initiation of ERT with alglucosidase alfa since most patients have FVC data within this time period and only a small percentage of patients had data reported many years after. Data for never-treated patients were not evaluated because the majority of Registry patients have received therapy with alglucosidase alfa. Additionally, there were insufficient pre-treatment data in the Registry to sufficiently power comparisons of respiratory function trends pre- versus post-ERT. The low data availability in the pre-treatment period may reflect enrollment in the Registry after ERT initiation and/or differences in the standard of care for performing FVC assessments during the pre-treatment period and the desire to treat patients promptly following diagnosis of Pompe disease. We did not have sufficient data for assessment of FVC in the supine position, maximum inspiratory pressure (MIP), or maximum expiratory pressure (MEP). The present study did not evaluate the potential impact of tobacco use on respiratory function decline. Among the 237 patients who had smoking history data entered in the Registry at baseline, only 8 (3.4%) specified ongoing tobacco use at baseline. Thus, further analysis by smoking status was not conducted. Finally, it has to be emphasized that asymptomatic patients were not included in this study, which was designed to longitudinally investigate respiratory function during ERT in symptomatic patients enrolled in the Pompe Registry. By definition, diagnosis of LOPD requires onset of symptoms ≤ 12 months of age without cardiomyopathy or symptom onset at > 12 months of age, which excludes patients without overt neurological symptoms.

### Statistical analyses

Descriptive analyses were conducted for patients’ demographics and clinical characteristics. Distributions of baseline FVC were examined by prognostic factors in univariable analyses. Prognostic factors included sex; ages at symptom onset, diagnosis, and ERT initiation; time from symptom onset to ERT initiation; time from diagnosis to ERT initiation; baseline FVC (dichotomized); respiratory and ambulatory support at baseline; and use of an ambulation device at baseline. Analyses to assess the slope of FVC over time among all patients and among subgroups of prognostic factors were conducted using a linear mixed-model regression, which accounts for correlations between multiple assessments within patients. Review of FVC data over the course of treatment and comparisons of different mixed models indicated that FVC generally followed a linear course. Thus, a linear mixed-model regression was considered appropriate. FVC between subgroups of prognostic factors, adjusting for age at first ERT, was calculated. The slope was interpreted as the change in FVC per year. Estimates and *p* values were obtained from linear mixed effect regression models fitted with FVC as the outcome. Each model included follow-up years, the prognostic factor, age at first ERT (if the parameter was not age), and one interaction term (prognostic factor x follow-up years). A non-significant slope indicated that FVC remained stable over time. FVC Difference Between Groups compared whether FVC was higher or lower for one group compared with another during the 5 years of follow-up. A negative difference indicated one group had a lower FVC than the other group designated as the reference. Additional details on the risk factors adjusted for are provided in the table and figure footnotes. Sensitivity analyses were conducted to assess the robustness of the results. Statistical analyses were performed using SAS statistical software version 9.4 (SAS Institute Inc., Cary, NC, USA). An alpha level of 0.05 was used as the criterion for statistical significance.

## Data availability

The data that support the findings of this study can be requested by Pompe Registry participants through a Pompe Registry Data Analyses Request form. The data are not publicly available due to privacy or ethical restrictions. For additional information, please contact rarediseaseregistries@sanofi.com.

## Results

### Patient disposition

Of the 1190 Registry patients with LOPD who were treated with alglucosidase alfa as of December 2018, 956 had valid FVC assessments. Of these, 543 had valid FVC assessments at ERT initiation (baseline for this analysis). The final analysis population comprised 396 patients who met all eligibility criteria; one patient was excluded for having an extreme slope value (≤ − 10%/year or ≥ + 10%/year). Given that not all patients with baseline data (*n* = 543) also had adequate longitudinal data, we assessed the generalizability of the final patient population. For the 543 patients, the median ages at symptom onset, diagnosis, and ERT initiation were 34.7 years, 41.4 years, and 45.2 years, respectively. Their median baseline FVC was 67.0% of predicted. These values are similar to those reported for the final population of 396 patients (see below and Table 1).

**Table 1:** Demographics and clinical characteristics for all patients and by Shorter-Time and Longer-Time groups^a^

	All patients (*N* = 396)	Shorter-Time Group (*N* = 198)	Longer-Time Group (*N* = 198)	*p* value^b^
Sex, *n*	396	198	198	0.62
Male, *n* (%)	198 (50.0)	96 (48.5)	102 (51.5)	
Female, *n* (%)	198 (50.0)	102 (51.5)	96 (48.5)	
Region, *n*	396	198	198	< 0.01
Europe, *n* (%)	288 (72.7)	129 (65.2)	159 (80.3)	
North America, *n* (%)	94 (23.7)	59 (29.8)	35 (17.7)	
Rest of World, *n* (%)^c^	14 (3.5)	10 (5.1)	4 (2.0)	
Race, *n*	396	198	198	0.08
Asian, *n* (%)	15 (3.8)	8 (4.0)	7 (3.5)	
Black, *n* (%)	3 (0.8%	1 (0.5)	2 (1.0)	
White, *n* (%)	336 (84.8)	160 (80.8)	176 (88.9)	
Multiple, *n* (%)	2 (0.5)	2 (1.0)	0	
Not reported, *n* (%)	2 (0.5)	2 (1.0)	0	
Unknown/missing, *n* (%)	38 (9.6)	25 (12.6)	13 (6.6)	
Age (years) at symptom onset, *n*	381	190	191	0.03
Median (25%, 75%)	33.7 (17.0, 45.0)	35.9 (20.4, 45.3)	31.8 (14.2, 43.5)	
Min, max	0.0, 73.7	0.0, 73.7	0.0, 68.1	
Age (years) at diagnosis, *n*	396	198	198	< 0.01
Median (25%, 75%)	41.1 (29.2, 53.1)	44.4 (34.1, 56.1)	38.4 (24.5, 50.6)	
Min, max	0.3, 80.6	5.8, 80.6	0.3, 77.4	
Age (years) at ERT initiation, *n*	396	198	198	0.66
Median (25%, 75%)	45.0 (34.8, 57.2)	45.1 (34.7, 56.4)	44.8 (34.9, 57.8)	
Min, max	5.9, 81.0	5.9, 81.0	6.0, 79.2	
Symptom onset to ERT initiation (years), *n*	358	174	184	< 0.01
Median (25%, 75%)	10.3 (4.4, 17.8)	8.0 (2.5, 14.9)	12.0 (7.9, 19.8)	
Min, max	0.0, 64.1	0.0, 59.7	0.0, 64.1	
Diagnosis to ERT initiation (years), *n*	396	198	198	< 0.01
Median (25%, 75%)	1.7 (0.4, 7.6)	0.4 (0.2, 0.7)	7.6 (3.5, 11.1)	
Min, max	0.0, 30.9	0.0, 1.7	1.8, 30.9	
Patients with at least 1 IVS1 variant, *n*	303	163	140	
Yes, *n* (%)	265 (87.5)	141 (86.5)	124 (88.6)	0.61
No, *n* (%)	38 (12.5)	22 (13.5)	16 (11.4)	
Baseline FVC value, *n*	396	198	198	0.09
Median (25%, 75%)	66.9 (49.0, 85.0)	68.1 (52.0, 86.2)	66.0 (47.0, 83.0)	
Min, max	9.3, 126.0	15.4, 126.0	9.3, 125.0	
Respiratory support at baseline, *n*	188	105	83	0.07
No, *n* (%)	158 (84.0%)	93 (88.6%)	65 (78.3%)	
Yes, *n* (%)	30 (16%)	12 (11.4%)	18 (21.7%)	
Type of respiratory support, *n*	30	12	18	
Supplemental oxygen, *n* (%)	1 (3.3%)	0	1 (5.6%)	
Non-invasive support, *n* (%)	29 (96.7%)	12 (100%)	17 (94.4%)	
If non-invasive support, duration of support, *n*	29	12	17	
< 24 h (night and day), *n* (%)	4 (13.8%)	2 (16.7%)	2 (11.8%)	
Night only, *n* (%)	23 (79.3%)	9 (75.0%)	14 (82.4%)	
Unknown, *n* (%)	2 (6.9%)	1 (8.3%)	1 (5.9%)	
Ambulatory at baseline, *n*	113	61	52	0.41
No, *n* (%)	6 (5.3%)	2 (3.3%)	4 (7.7%)	
Yes, *n* (%)	107 (94.7%)	55 (96.7%)	48 (92.3%)	
Use of ambulation devices at baseline, *n*	187	107	80	< 0.01
No, *n* (%)	137 (73.3%)	90 (84.1%)	47 (58.8%)	
Yes, *n* (%)	50 (26.7%)	17 (15.9%)	33 (41.3%)	
Type of ambulation device				
Wheelchair, *n* (%)	24 (48.0%)	4 (23.5%)	20 (60.6%)	
Walker, *n* (%)	5 (10.0%)	3 (17.6%)	2 (6.1%)	
Cane, *n* (%)	19 (38.0%)	10 (58.8%)	9 (27.3%)	
Other, *n* (%)	2 (4.0%)	0	2 (6.1%)	

Shorter-Time group includes patients with a time from diagnosis of Pompe disease to initiation of ERT with alglucosidase alfa ≤ the median exposure (0–1.7 years); Longer-Time group includes patients with a time from diagnosis of Pompe disease to initiation of ERT with alglucosidase alfa > the median exposure (> 1.7 years)

*ERT *enzyme replacement therapy, *Max *maximum, *Min *minimum, *SD *standard deviation

^a^Denominators of percentages were calculated from the data available for each parameter

^b^*p* value tested if the Shorter-Time vs Longer-Time Groups were different by the characteristics shown. *p* values were obtained from Wilcoxon test (continuous variables) or Fisher's exact test (categorical variables)

^c^Patients from Latin America, the Asia Pacific, and the Middle East were combined due to small numbers

### Demographics

Demographics for the 396 patients are provided in Table 1. Males and females were evenly distributed (*n* = 198 for both). Most patients were from Europe (72.7%) or North America (23.7%). Median ages at symptom onset, diagnosis, and ERT initiation were 33.7 years, 41.1 years, and 45.0 years, respectively. The median duration of time from symptom onset to ERT initiation was 10.3 years (interquartile range [IQR]: 4.4–17.8 years). The median time from Pompe disease diagnosis to ERT initiation was 1.7 years (IQR: 0.4–7.6 years). Among 303 patients with variant data, 265 patients (87.5%) had at least one c.-32-13 T > G (IVS1) allele. There were 102 patients (25.8%) who reported having siblings diagnosed with Pompe disease (data not shown).

### Baseline clinical characteristics

Table 1 also describes patient baseline clinical characteristics. The median baseline FVC measurement was 66.9% predicted (IQR 49.8–85.0%). Among the 30 patients on respiratory support at baseline, 29 were on non-invasive support (of whom 79.3% were on support during the night only) and 1 used supplemental oxygen. Among the 50 patients who used an ambulation device at baseline, 24 had reported wheelchair use.

### Longitudinal change in FVC during ERT

**Fig. 1:**
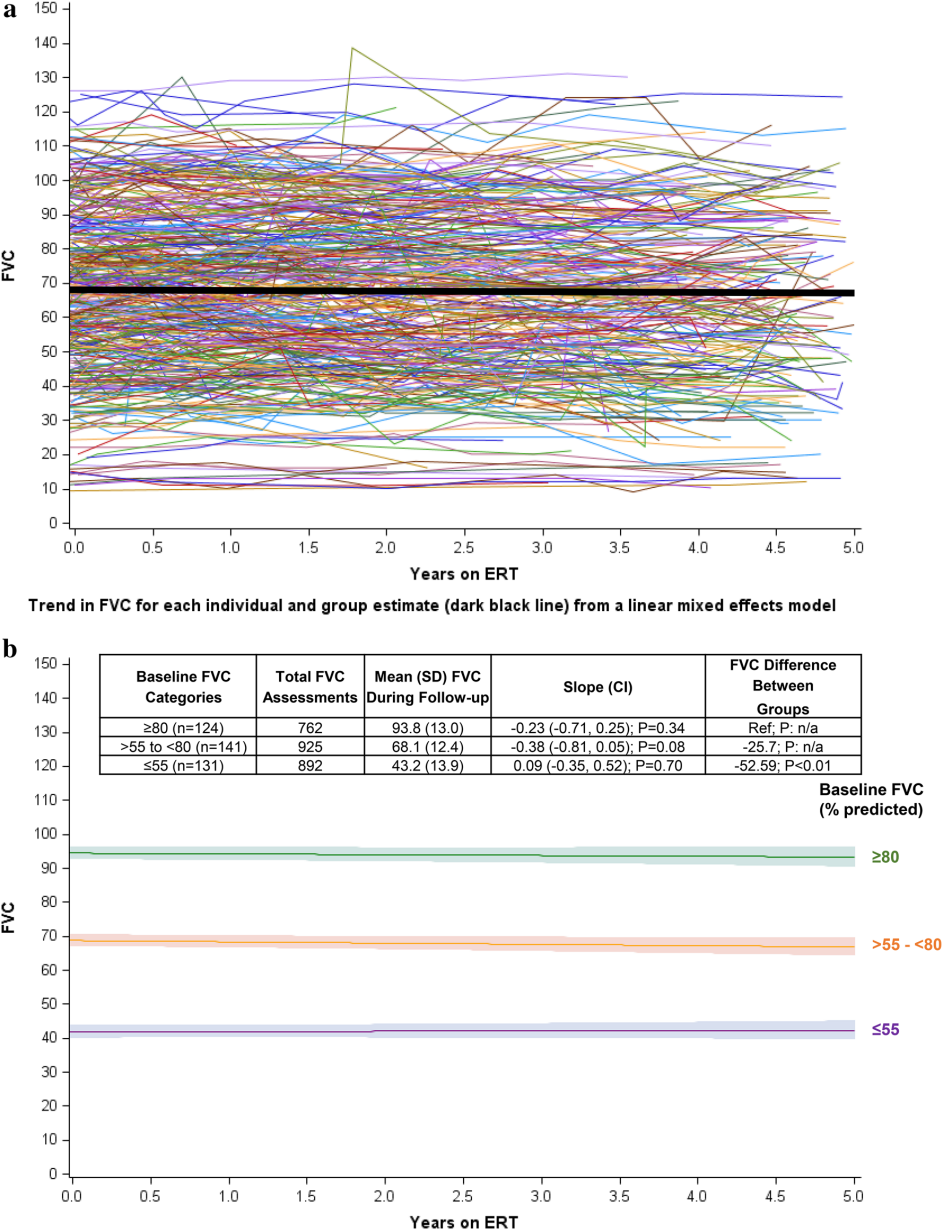
FVC during ERT with alglucosidase alfa over time. **a** Percent predicted FVC in the upright position among all patients (*N* = 396). Individual lines represent each individual patient's FVC values plotted over time longitudinally. The thick dark line is the estimate of all patients from the mixed model adjusted for age at first ERT. The median follow-up time for all patients was 4.0 years (range 0.5–5.0; interquartile range 2.7–4.6). **b** Percent predicted FVC in the upright position by baseline FVC. FVCs for patients grouped by different baseline FVC classifications. Each line corresponds to patients classified into one of three groups by their baseline FVC %predicted as follows: ≤ 55%predicted; > 55 to < 80%predicted; or ≥ 80%predicted). Each line is the estimate from the mixed model adjusted for age at ERT initiation with their confidence intervals (shaded area around line). The group of patients with a baseline FVC ≥ 80 was the reference group. *CI *confidence interval, *ERT *enzyme replacement therapy, *FVC *percent (%) predicted forced vital capacity in the upright position, *SD *standard deviation

Figure 1a shows FVC during the course of up to 5 years on ERT among all 396 patients. Median follow-up time was 4.0 years (range 0.5–5 years; IQR 2.7–4.6 years) and many of the patients underwent FVC assessments during most of the 5-year period. The median number of FVC assessments per patient during follow-up was 5 (range 2–16 assessments). In total, 2579 FVC assessments were analyzed. The slope for change in FVC over time was − 0.17% of predicted/year (CI − 0.42, 0.09), which is not statistically different from zero (*p* = 0.21), indicating that, on average, FVC remained stable during ERT.

Figure 1b compares FVC during ERT among the 396 patients when grouped into one of three groups classified by severity of baseline respiratory dysfunction: ≤ 55% predicted (*n* = 131); > 55 to < 80% predicted (*n* = 141); and ≥ 80% predicted (*n* = 124). As shown, FVC remained stable during ERT in all three groups (slopes: ≤ 55, 0.09 [CI − 0.35, 0.52; *p* = 0.70]; > 55 to < 80, − 0.38 [CI − 0.81, 0.05; *p* = 0.08]; ≥ 80, − 0.23 [CI − 0.71, 0.25; *p* = 0.34]).

### Prognostic factors of FVC at baseline and follow-up

Given the heterogeneity of Pompe disease, we assessed the relationship between various prognostic factors for FVC at baseline (Table 2). Baseline FVC was lower among males compared with females. Age at symptom onset was not associated with degree of respiratory dysfunction at baseline. In contrast, respiratory function was more impaired in patients who were older at ERT initiation (> median age of 45.0 years; IQR = 34.8–57.2 years) and patients with a longer duration from either symptom onset or diagnosis to ERT initiation. In addition, individuals with advanced disease at baseline based on either use of respiratory support, inability to ambulate, or ambulation device use demonstrated the greatest degree of respiratory dysfunction.

**Table 2:** FVC at baseline for all patients and patients grouped by prognostic factors^a^

Prognostic factor	Patients (*N*)	Mean (SD) baseline FVC	Interquartile range of baseline FVC (25%, 75%)	*p* value^b^
All Patients	396	67.0 (24.2)	49.8, 85.0	n/a
Sex				< 0.01
Male	198	60.8 (24.6)	43.5, 77.0	
Female	198	73.2 (22.3)	58.0, 89.3	
Age (years) at symptom onset				0.93
≤ 12	67	66.9 (24.1)	49.0, 82.0	
> 12	314	66.7 (24.2)	49.5, 85.0	
Age (years) at symptom onset				0.70
< 18	103	65.2 (24.8)	49.0, 82.0	
≥ 18	278	67.3 (24.0)	50.0, 85.0	
Age (years) at symptom onset				0.73
≤ Median (33.7)	191	66.7 (26.2)	46.6, 87.0	
> Median	190	66.9 (22.0)	51.0, 82.0	
Age (years) at diagnosis				0.05
≤ Median (41.1)	198	68.8 (26.8)	49.0, 89.0	
> Median	198	65.2 (21.2)	50.3, 79.3	
Age (years) at ERT initiation				< 0.01
≤ Median (45.0)	198	71.0 (26.4)	53.0, 90.0	
> Median	198	63.0 (21.2)	48.3, 76.6	
Symptom onset to ERT initiation (years)				0.01
≤ Median (10.3)	179	69.6 (24.9)	49.5, 89.0	
> Median	179	62.4 (22.7)	48.0, 78.0	
Diagnosis to ERT initiation (years)				0.09
≤ Median (1.7)	198	69.4 (23.1)	52.0, 86.2	
> Median	198	64.7 (25.2)	47.0, 83.0	
Baseline FVC, Categories				< 0.01
≤ Median (66.9)	198	47.1 (14.1)	37.4, 58.0	
> Median	198	86.9 (13.4)	76.0, 96.0	
Respiratory support at baseline^c^				< 0.01
No	158	78.4 (18.8)	65.0, 91.0	
Yes	30	51.8 (19.9)	37.4, 65.0	
Ambulatory at baseline^c^				0.01
No	6	33.1 (26.5)	14.0, 66.0	
Yes	107	68.2 (22.6)	51.0, 86.0	
Use of ambulation device at baseline^c^				< 0.01
No	137	73.8 (22.1)	57.7, 90.0	
Yes	50	58.5 (24.3)	39.7, 76.0	

*ERT *enzyme replacement therapy, *FVC *percent (%) predicted forced vital capacity in the upright position, *SD *standard deviation, *n/a *not applicable

^a^See “Methods” for definition of baseline. Values come from descriptive statistics

^b^*p* value tested if the baseline FVC values were different by the categories of the prognostic factor. *p* values were obtained from Wilcoxon test

^c^This status may be due to patients enrolling in the Pompe Registry after initiation of ERT and/or the differences in regional and clinical practices at Registry sites

The differences in FVC at baseline (Table 2) remained during follow-up (Table 3). The slopes over time were not statistically different from zero across most prognostic factors, indicating stability in respiratory function (Table 3). An exception was that there was a marginal decline over time among patients with older age of symptom onset. Patients > 12 years of age at symptom onset (*n* = 314) had a slope of − 0.31% of predicted per year (*p* = 0.04). Patients ≥ 18 years of age at symptom onset (*n* = 278) had a slope of − 0.34% of predicted per year (*p* = 0.03). Patients older than 33.7 years, the median age at symptom onset (*n* = 190), had a slope of − 0.40% of predicted per year (*p* = 0.04). No significant decline in FVC was observed in the comparator groups for each categorization at symptom onset (≤ 12 years [*n* = 67]: slope = 0.35% of predicted/year, *p* = 0.26; < 18 years [*n* = 103]: slope = 0.20% of predicted/year, *p* = 0.43) or ≤ median age at symptom onset [*n* = 191]: slope = 0.02% of predicted/year, *p* = 0.92).

**Table 3:** FVC during follow-up for all patients and patients grouped by prognostic factors^a^

Prognostic factor	Patients (*N*)	Total FVCs at baseline and follow-up (*N*)	Slope for each prognostic factor group^b^	*p* value of slope^b^	FVC difference between groups^c^	*p* value of FVC difference^c^
All patients	396	2579	− 0.17	0.21	n/a	n/a
Sex						
Male	198	1359	− 0.19	0.29	− 12.73	< 0.01
Female	198	1220	− 0.14	0.47	ref	n/a
Age (years) at symptom onset						
≤ 12	67	476	0.35	0.26	1.3	0.68
> 12	314	2016	− 0.31	0.04	ref	n/a
Age (years) at symptom onset						
< 18	103	713	0.20	0.43	− 1.04	0.70
≥ 18	278	1779	− 0.34	0.03	ref	n/a
Age (years) at symptom onset						
≤ Median (33.7)	191	1273	0.02	0.92	0.58	0.81
> Median	190	1219	− 0.40	0.04	ref	n/a
Age (years) at diagnosis						
≤ Median (41.1)	198	1320	− 0.03	0.85	3.91	0.10
> Median	198	1259	− 0.31	0.11	ref	n/a
Age (years) at ERT initiation						
≤ Median (45.0)	198	1294	0.02	0.91	8.59	< 0.01
> Median	198	1285	− 0.36	0.06	ref	n/a
Years from symptom onset to ERT initiation						
≤ Median (10.3)	179	1195	− 0.08	0.69	6.49	0.01
> Median	179	1143	− 0.28	0.15	ref	n/a
Baseline FVC						
≤ Median (67.0)	198	1327	− 0.11	0.54	− 37.7	< 0.01
> Median	188	1252	− 0.22	0.24	ref	n/a
Respiratory support at baseline						
No	158	951	− 0.16	0.44	22.85	< 0.01
Yes	30	206	0.29	0.52	ref	n/a
Ambulatory at baseline						
No	6	31	1.26	0.21	− 36.07	< 0.01
Yes	107	720	− 0.07	0.76	ref	n/a
Use of ambulatory device at baseline						
No	137	832	− 0.09	0.65	13.5	< 0.01
Yes	50	359	− 0.55	0.09	ref	n/a

*ERT *enzyme replacement therapy, *FVC *percent (%) predicted forced vital capacity in the upright position, *n/a *not applicable, *ref *reference

^a^See “Methods” for definition of baseline

^b^The slope is interpreted as the change in FVC per year. Estimates and *p* values were obtained from linear mixed effect regression models fitted with FVC as the outcome. Each model included follow-up years, the prognostic factor in the first column, age at first ERT (if the parameter was not age), and one interaction term (prognostic factor*follow-up years). A non-significant slope indicated that FVC remained stable

^c^FVC Difference Between Groups column compared whether FVC was higher or lower for one group compared with another during the 5 years of follow-up. A negative difference indicated one group had a lower FVC than the other group designated as the reference

### Impact of Shorter-Time vs. Longer-Time from diagnosis to initiation of ERT on respiratory function

**Fig. 2:**
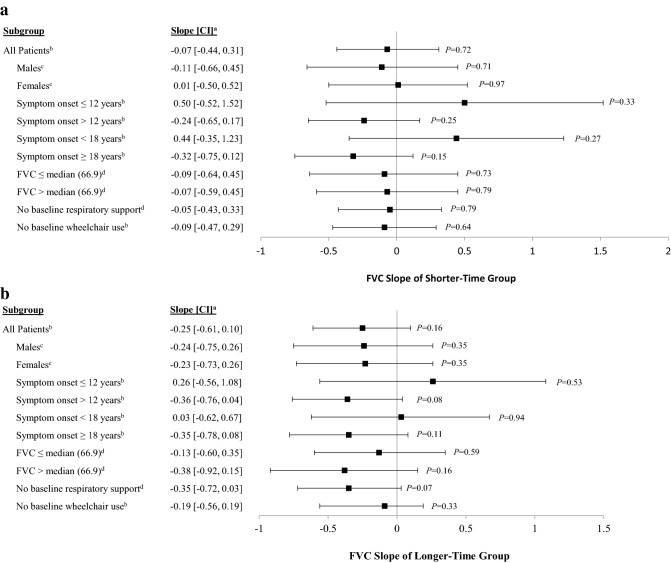
Slopes of FVC for the Shorter-Time^a^ and Longer-Time^a^ groups among all patients and by subgroups. **a** Slopes of FVC for the Shorter-Time group. **b** Slopes of FVC for the Longer-Time group. ^a^Shorter-Time group includes patients with a time from diagnosis of Pompe disease to initiation of ERT with alglucosidase alfa ≤ the median (0–1.7 years); Longer-Time group includes patients with a time from Pompe diagnosis to initiation of ERT with alglucosidase alfa > the median (> 1.7 years). Estimates and *p* values were obtained from separate linear mixed effect regression models fitted with FVC as the outcome. Each model included years of follow-up, Shorter- vs. Longer-Time groups, and an interaction term between the Shorter- vs. Longer-Time groups and years of follow-up. Models are slightly different depending on the subgroups to avoid over-adjusting. Thus, further adjustments are described below. ^b^The mixed model was further adjusted for sex, baseline FVC, and age at ERT initiation. ^c^The mixed model was further adjusted for baseline FVC and age at ERT initiation. ^d^The mixed model was further adjusted for sex and age at ERT initiation. *ERT *enzyme replacement therapy; *FVC *percent (%) predicted forced vital capacity in the upright position, *CI *confidence interval; *P* is *P* value.

The slopes of FVC change over time for patients in the Shorter-Time (Fig. 2a) and Longer-Time (Fig. 2b) groups were assessed. The slopes for change in FVC during up to 5 years since ERT initiation were not statistically different from zero for either group (Shorter-Time: − 0.07% predicted/year, *p* = 0.72; Longer-Time: − 0.25% predicted/year, *p* = 0.16), indicating that respiratory function remained stable during the course of treatment for both groups. Similarly, the FVC slopes among patients in both the Shorter- and Longer-Time groups were not statistically significantly different when subjects were grouped by different baseline characteristics (Fig. 2a, b).

**Fig. 3:**
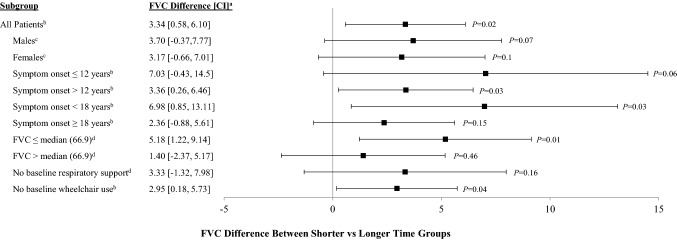
FVC difference between Shorter-Time^a^ versus Longer-Time^a^ groups for all patients and by subgroup populations. ^a^Shorter-Time group includes patients with a time from diagnosis of Pompe disease to initiation of ERT with alglucosidase alfa ≤ the median (0–1.7 years); Longer-Time group includes patients with a time from diagnosis of Pompe disease to initiation of ERT with alglucosidase alfa > the median (> 1.7 years). Estimates and *p* values were obtained from separate linear mixed effect regression models fitted with FVC as the outcome. Each model included years of follow-up, Shorter- vs. Longer-Time groups, and an interaction term between the Shorter- vs. Longer-Time groups and years of follow-up. Models are slightly different depending on the subgroups to avoid over-adjusting. Thus, further adjustments are described below. The *p* value tested if the FVC difference between Shorter-Time vs. Longer-Time groups was statistically different. ^b^The mixed model was further adjusted for sex, baseline FVC, and age at ERT initiation. ^c^The mixed model was further adjusted for baseline FVC and age at ERT initiation. ^d^The mixed model was further adjusted for sex and age at ERT initiation. *ERT *enzyme replacement therapy, *FVC *percent (%) predicted forced vital capacity in the upright position, *CI* confidence interval, *P* is the P value

We also assessed whether there were differences between Shorter-Time compared to Longer-Time with respect to FVC over time. Among all patients, FVC was significantly higher in the Shorter-Time versus Longer-Time group (Fig. 3). The difference in FVC between the Shorter-Time versus Longer-Time groups was 3.34% predicted (*p* = 0.02), adjusting for prognostic factors. Among different subgroups of patients (Fig. 3) in general, we still saw an indication of benefit of shorter time from diagnosis to treatment in each subgroup. This was even observed among patients who started with an FVC ≤ median of 66.9% predicted, with patients in the Shorter-Time group having an FVC that was 5.18% higher than that in the Longer-Time group. Some estimates tended to be higher than others, particularly among patients who were younger at their ages of symptom onset (using both definitions of ≤ 12 [7.03% higher in the Shorter-Time group] and < 18 years of age [6.98% higher in the Shorter-Time group]).

### Sensitivity analyses

Sensitivity analyses were conducted to evaluate whether the observed difference in FVC during ERT in the Shorter- and Longer-Time groups would vary using other cut-offs for the definition of exposure (time from diagnosis to ERT initiation). When the lowest versus highest tertiles were compared, the estimated difference in baseline FVC between the groups was larger at 4.77% predicted (*p* < 0.01); this difference persisted during follow-up. Results also remained significant when assessing the exposure continuously. For that model, a 1-year decrease in exposure (time from diagnosis to ERT start) was associated with an increase in baseline FVC of 0.58% predicted, which was statistically significant (*p* < 0.01).

Comparison of the Shorter-Time group compared to the Longer-Time group indicated that patients were similar with respect to sex, race, age at ERT initiation, IVS1 frequency, use of respiratory support at baseline, and being ambulatory at baseline (Table 1). As expected, there were differences in age at diagnosis and age at symptom onset since these are related to the exposure definition. Of note, patients in the Shorter-Time group were more likely to be able to ambulate without using an ambulation device at baseline when compared to the Longer-Time group (84.1% vs. 58.8%). Furthermore, excluding patients with no baseline wheelchair use still indicated a benefit among the Shorter-Time group with an FVC difference of 2.95% (*p* = 0.04) (Fig. 3).

### Respiratory support

Initiation of respiratory support during the longitudinal follow-up period was assessed. Among the 158 patients who had reported data at baseline and also were reported as not receiving respiratory support at baseline, 26 (16.5%) initiated respiratory support during the 5-year follow-up (Table 4). The number of male (*n* = 12) vs. female (*n* = 14) patients was similar. In this subgroup, median age at symptom onset was 30.5 years, and median time from symptom onset to diagnosis was 18.8 years. The median baseline FVC for these patients was 65.5% predicted. No patients started invasive respiratory support during follow-up. Most (92.3%; *n* = 24) went on non-invasive support and during night-time only (91.7%; *n* = 22); 1 (3.8%) patient was using supplemental oxygen, and the type of respiratory support was not reported in the Registry for 1 (3.8%) patient.

**Table 4:** Characteristics of patients who initiated respiratory support during follow-up

Characteristic	Result
Patients who initiated respiratory support during follow-up^a^, *n* (%)	26/158^b^ (16.5%)
Incidence of first use of respiratory support per 1000 patient years^c^	42.9
Males/females, *n* (%)	12 (46.2%)/14 (53.8%)
Age (years) at first use of respiratory support^d^, *n*	26
Mean (SD)	48.3 (20.2)
Median (25%, 75%)	49.9 (33.9, 62.4)
Min, Max	9.9, 79.7
Type of support at first use of respiratory support, *n*	26
Invasive support, *n* (%)	0
Non-invasive support, *n* (%)	24 (92.3%)
Duration of non-invasive support, *n* (%)	
24 h/day	0
< 24 h (night and day)	2 (8.3%)
Night only	22 (91.7%)
Unknown	0
Supplemental oxygen, *n* (%)	1 (3.8%)
Unknown type, *n* (%)	1 (3.8%)
Baseline FVC^d^, *n*	26
Mean (SD)	67.7 (19.4)
Median (25%, 75%)	65.5 (55.0, 80.0)
Min, Max	35.0,123.0
Age at symptom onset (years)^d^, *n*	25
Mean (SD)	28.6 (16.7)
Median (25%, 75%)	30.5 (13.6, 36.7)
Min, Max	0.1, 60.7
Time from symptom onset to diagnosis (years)^d^, *n*	22
Mean (SD)	19.1 (14.8)
Median (25%, 75%)	18.8 (7.0, 30.1)
Min, Max	0.5, 51.8
Time from symptom onset to ERT initiation (years)^d^, *n*	24
Mean (SD)	20.0 (15.7)
Median (25%, 75%)	18.7 (8.9, 31.0)
Min, Max	0.0, 56.7
Time from diagnosis to ERT initiation (years)^d^, *n*	26
Mean (SD)	2.4 (3.6)
Median (25%, 75%)	0.5 (0.3, 2.7)
Min, Max	0.0, 12.8
Use of ambulation devices at baseline^d^, *n*	21
No, *n* (%)	17 (81.0%)
Yes, *n* (%)	4 (19%)
Type of ambulation device	
Wheelchair, n (%)	2 (50.0%)
Walker, *n* (%)	0
Crutches, *n* (%)	0
Cane, *n* (%)	2 (50.0%)
Other, *n* (%)	0
Unknown,* n* (%)	0

*ERT *enzyme replacement therapy, *FVC *percent (%) predicted forced vital capacity in the upright position, *SD *standard deviation

^a^Calculated among patients who had not initiated respiratory support in baseline period of − 180/ + 28 days of ERT. See Methods for further information on how baseline was defined

^b^The 158 patients who had reported data at baseline and also were reported as not receiving respiratory support at baseline

^c^Incidence of first use of respiratory support per 1000 patient years was calculated by taking the number patients who initiated respiratory support during follow-up and dividing this number by the total follow-up time in years. This number was multiplied by 1000

^d^Among 26 patients who initiated respiratory support during follow-up

## Discussion

This study was designed to evaluate the course of respiratory function during ERT with alglucosidase alfa, prognostic factors for FVC, and the impact of time from diagnosis to initiation of therapy. The data indicate that respiratory function, assessed by upright FVC % predicted, remained stable over the 5-year follow-up for Registry patients with LOPD. These results extend the findings of previous studies, including the Dutch study of 102 adult LOPD patients [9], the Late-Onset Treatment Study (LOTS) [8], and the LOTS extension study [7] by studying a large global patient cohort over a long follow-up duration. In our analysis, there were only 26 patients who initiated respiratory support during ERT with alglucosidase alfa, further suggesting stability of respiratory status during treatment. This result also contrasts with the data published by Hagemans et al., who found an 8% increase in the odds for respiratory support for each year untreated after diagnosis in a cohort of LOPD patients [12]. However, we also must consider that other factors may play a role in the need for respiratory support, and the decision to implement non-invasive ventilation is not based solely on FVC assessments and may reflect results of arterial blood-gas analysis and/or sleep studies [13].

Because Pompe disease presents as a wide disease spectrum, data for separate subgroups of patients were analyzed. These analyses indicated that FVC remained stable over time among different subgroups, except for patients with an older age of symptom onset.

The observation that respiratory function remains stable during ERT in our study contrasts with observations of longitudinal trends in untreated patients and has implications for the long-term prognosis in LOPD. Prior studies of untreated LOPD patients reported progressive decline in FVC ranging from 1.0 to 4.6%/year [14–18]. Stabilization of respiratory function during ERT with alglucosidase alfa was demonstrated initially during 78 weeks of treatment in the LOTS trial [8]. Longer-term stability in respiratory function was suggested in other studies that reported results during up to 5 years of ERT [7, 9, 19, 20]. Results of our study confirm and extend these findings in the largest global population of Pompe patients followed in real-world clinical settings.

The nature and extent of data in the Pompe Registry also allowed us to evaluate the impact of time between diagnosis of LOPD and ERT initiation on respiratory function. The longitudinal data revealed stability of respiratory function in both the Shorter-Time and Longer-Time groups during 5 years of follow-up. Nevertheless, for the subjects studied, earlier initiation of ERT with alglucosidase alfa (i.e., in a shorter time after diagnosis) is associated with higher FVC at baseline that persists over time compared to treatment initiation following a longer time after diagnosis across the disease spectrum. This finding suggests that the sooner ERT is initiated after diagnosis, the better the long-term outcome is with respect to respiratory function.

It is also important to note that respiratory function also remained stable in the patients with a longer time from diagnosis to treatment initiation (i.e., Longer-Time group). Although onset and progression of Pompe disease are variable, a longer delay between diagnosis and treatment generally tends to be associated with more advanced disease [14, 21]. Nevertheless, in our analysis, stability of respiratory function was observed in the Longer-Time group despite the delay in initiating ERT in these patients.

The stability of respiratory function observed in this analysis is clinically meaningful for numerous reasons. Most notably, respiratory morbidity in patients with neuromuscular disorders, including Pompe disease, is closely linked to the degree of muscle weakness and the resulting level of respiratory dysfunction. For example, the development of respiratory failure and requirement for non-invasive ventilation or tracheostomy can be predicted based on an individual patient’s FVC [22–26]. Once respiratory support is initiated, there is a predictable reduction in physical function on patient-reported outcome surveys [27]. Of importance, in our cohort of patients, respiratory function was stable, and initiation of non-invasive ventilation was observed in only a few individuals during the duration of follow-up. These findings parallel those reported recently demonstrating that patients on ERT with alglucosidase alfa still showed a better motor outcome after 5 years of ERT compared to the start of ERT, and that ERT is associated with sustained improvement in skeletal muscle function [19]. Taken together, these beneficial effects of ERT on muscle function in patients with Pompe disease may explain earlier suggestions of reduced mortality among patients receiving ERT [19, 28]. These findings suggest that additional longitudinal analyses of LOPD patients would reveal reduction in respiratory morbidity and possibly mortality during ERT as was demonstrated in patients with the more rapidly progressive infantile-onset form of Pompe disease [29]. While beyond the scope of the analysis reported here, such analyses of Pompe Registry data are planned for the future.

### Study considerations

It is challenging to reliably perform FVC tests in the real-world setting compared with a controlled trial. This variation would likely introduce random misclassification of FVC that could attenuate any differences between the groups. Additionally, there are limitations to applying a linear mixed model, which assumes a linear slope of FVC over time, to observational data that are not derived from regular clinical visits, resulting in a variable number of data points and uneven time intervals between FVC assessments. However, the results shown in Fig. 1a do not suggest a change in the slope of FVC during the follow-up period. Qualitatively, the linearity assumption does not appear to be violated. To account for implausible values, outliers (patients with values > 150% predicted and patients with slopes ≤ − 10% and ≥ 10% /year) were excluded. FVC can be challenging to reliably administer in very young children and thus, only patients aged ≥ 5 years were analyzed. In addition, although assessment of diaphragm function could be performed by analysis of supine vital capacity, there were insufficient data available in the Registry, which likely reflects variability in clinical practice across Registry sites. While sites are encouraged to enter all available longitudinal data, incomplete reporting is possible and could underestimate the data availability. Lastly, while respiratory muscle strength can be directly assessed by inspiratory and expiratory muscle pressures (MIP and MEP, respectively), these measurements were not included in the present study based on insufficient available data and their variability both within and across visits in any given individual [30, 31].

Although the analyses were adjusted, there still may be residual differences between groups when testing the association between the Shorter-Time and Longer-Time groups and FVC (confounding). Sensitivity analyses were conducted to assess the robustness of the results, which were generally consistent with the main result. There was no “control” comparator, e.g., never on ERT or pre-ERT comparator as there were in other studies. Generally, declines in FVC and general clinical status have been reported in previous studies in patients who were never on alglucosidase alfa or in those that compare pre- and post-treatment results [7, 8, 18]. Additionally, as shown in Table 1, patients in the Shorter-Time group were older than those in the Long-Time group at symptom onset (median: 35.9 vs. 31.8 years) and at diagnosis (44.4 vs. 38.4 years). This could imply milder disease (that is, not clinically evident earlier) with slower progression in the Shorter-Time group, and that these patients would have done better even if there was a longer time between diagnosis and treatment initiation. Sensitivity analyses beyond the scope of our analysis here would be needed to confirm this. Also, patients in the Shorter-Time group were reported to be less likely to be using ambulatory assistance at baseline. Again, this could imply that the patients were in better physical condition and this was responsible for the better respiratory status reported for these patients.

The Pompe Registry represents the largest repository of data for Pompe disease. The Registry is an ongoing study with new patients being enrolled continually throughout the years. Therefore, longitudinal FVC data covering 5 years or more are unavailable for patients more recently enrolled. With the addition of more data over time, further analyses will be possible and will provide additional insights.

### Strengths of this analysis

Data from the Pompe Registry provide unique opportunities to evaluate and compare real-world data from patients at different sites around the world. This is the largest and one of the longest studies to date of FVC among treated Pompe disease patients. Data from 396 LOPD patients and nearly 2600 FVC assessments during a 5-year follow-up were analyzed. Most patients had multiple years of follow-up during this period (median: 4 years, with 25% of patients with a follow-up from 4.6–5.0 years). Patients started with a wide range of baseline FVCs, so it is unlikely that a select group of healthy patients were assessed. Availability of a large dataset also provides the opportunity to perform robust subgroup and sensitivity analyses. An indication of benefit of initiating ERT early on respiratory function persisted in subgroups defined by sex, age at symptom onset, and baseline FVC. In addition, while the primary analysis of shorter versus longer time was based on the median time from diagnosis to ERT initiation, sensitivity analyses examining time as tertiles or continuous also indicated statistically significant benefit of shorter delay to ERT initiation. Analyses comparing the final patient population (*n* = 396) who had adequate longitudinal data with patients who had baseline FVC assessments (*n* = 543) showed that patient characteristics were similar, indicating that results are generalizable. Lastly, exclusion of subjects who required ventilator support or wheelchair use at baseline did not affect results.

## Summary

Respiratory function remains stable in LOPD patients treated with alglucosidase alfa for up to 5 years. Reducing the time of initiation of ERT with alglucosidase alfa after diagnosis has a favorable impact of stabilizing FVC at a higher level. While the results of the present study indicate that delaying the start of ERT in symptomatic patients may result in less effective preservation of respiratory function over time, they are not per se suitable to guide the clinical decision in whom or when ERT should be started. Rather, the present findings strongly support the need to start ERT early once diagnosis and treatment indication have been established in order to stabilize respiratory function and prevent further decline. To this aspect, this study’s results are reinforced by prior studies that demonstrated stable or improved respiratory function in patients with advanced LOPD [13, 32, 33]. Analyses in larger cohorts over longer time frames will be facilitated as enrollment in the Pompe Registry increases and as additional longitudinal data are collected.
